# Dopamine Circuit Mechanisms of Addiction-Like Behaviors

**DOI:** 10.3389/fncir.2021.752420

**Published:** 2021-11-09

**Authors:** Carli L. Poisson, Liv Engel, Benjamin T. Saunders

**Affiliations:** ^1^Department of Neuroscience, University of Minnesota, Minneapolis, MN, United States; ^2^Medical Discovery Team on Addiction, University of Minnesota, Minneapolis, MN, United States; ^3^Graduate Program in Neuroscience, University of Minnesota, Minneapolis, MN, United States

**Keywords:** dopamine, striatum, addiction, substance use disorder, animal model, nigrostriatal, mesostriatal

## Abstract

Addiction is a complex disease that impacts millions of people around the world. Clinically, addiction is formalized as substance use disorder (SUD), with three primary symptom categories: exaggerated substance use, social or lifestyle impairment, and risky substance use. Considerable efforts have been made to model features of these criteria in non-human animal research subjects, for insight into the underlying neurobiological mechanisms. Here we review evidence from rodent models of SUD-inspired criteria, focusing on the role of the striatal dopamine system. We identify distinct mesostriatal and nigrostriatal dopamine circuit functions in behavioral outcomes that are relevant to addictions and SUDs. This work suggests that striatal dopamine is essential for not only positive symptom features of SUDs, such as elevated intake and craving, but also for impairments in decision making that underlie compulsive behavior, reduced sociality, and risk taking. Understanding the functional heterogeneity of the dopamine system and related networks can offer insight into this complex symptomatology and may lead to more targeted treatments.

## Introduction

**FIGURE 1:**
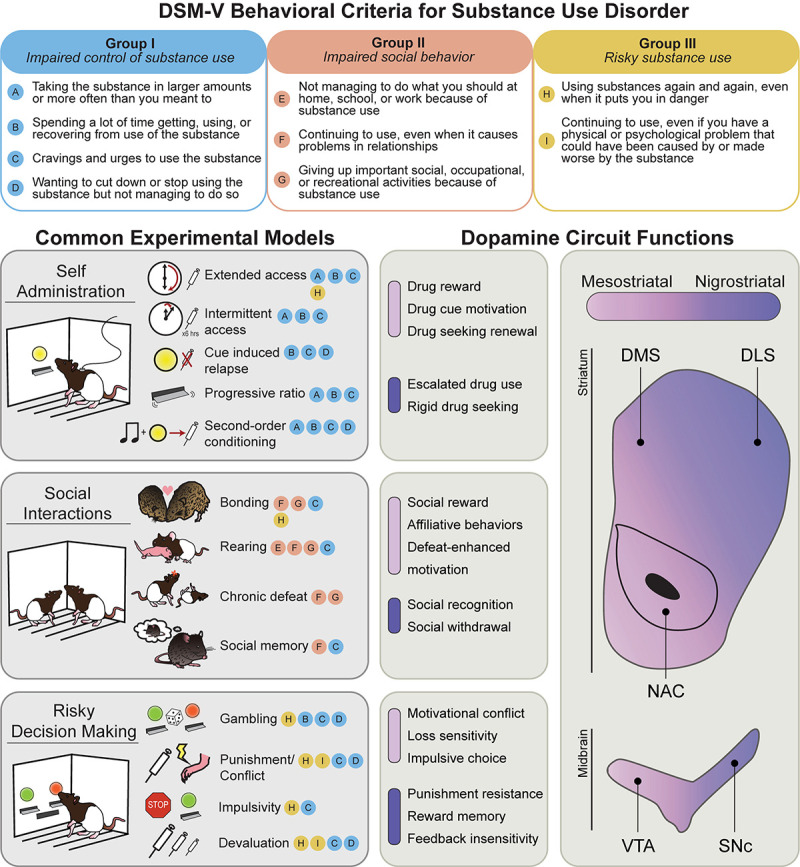
Behavioral models used to classify phenotypes of substance use disorder. **(Top)** The behavioral criteria of SUDs (circled letters) can be sorted into three main categories: impaired control of substance use (Group I), impaired social behavior (Group II), and risky substance use (Group III). **(Left)** Common rodent experimental models and the SUD criteria they are thought to best approximate. Note that most models capture multiple SUD features. **(Right)** Mesostriatal circuits (light purple), including dopamine projections from the ventral tegmental area (VTA) to the nucleus accumbens (NAC), and nigrostriatal circuits (dark purple), including dopamine projections to the dorsomedial (DMS) and dorsolateral striatum (DLS), have generally dissociable roles in different components of major SUD models. In the middle panels, the most clearly defined roles for these two systems in each SUD category are listed.

Addiction is characterized by a transition from recreational drug use to compulsive, disordered use, punctuated by cycles of abstinence, withdrawal, craving, and relapse. Features of human drug use are complicated by social and political factors, including stigmatization, criminalization, and barriers to treatment access. Over the past 30 years, the prevailing scientific consensus has identified addiction as a chronic disease, codified as substance use disorder (SUD). SUDs are characterized by pharmacological effects of tolerance and withdrawal, as well as a core set of behavioral features defined by the Diagnostic and Statistical Manual of Mental Disorders (DSM-5). These can be grouped into three major categories: I. Impaired control of substance use; II. Social impairment; and III. Risky use of substance (Figure 1, top). Significant research efforts have been made to characterize the neurobiological and psychological underpinnings of these behavioral symptoms. The hope is that understanding the basic science behind these behaviors will lead to more effective treatments for SUD, and other psychiatric illnesses with comorbid symptoms (such as compulsive gambling, ADHD, and schizophrenia).

Research making use of non-human animals is essential to this effort. Leveraging convergent biology of reward learning and decision-making systems across species, addiction scientists have established a variety of animal models to investigate drug-related behaviors (Figure 1). While considerable debate exists surrounding the translational efficacy of individual models to the complexity of human addiction (for recent review, see Venniro et al., 2020), they nonetheless offer powerful experimental insight into neurobehavioral mechanisms that govern core features of drug use.

Among brain systems, dopamine (DA) circuits are a key modulator of behaviors associated with SUDs. *Via* several mechanisms, including direct excitation of DA neurons (nicotine, alcohol), blockade of terminal DA reuptake (amphetamine, cocaine), and DA neuron disinhibition (opioids and cannabinoids), nearly all drugs used by humans acutely increase signaling of DA within the striatum, and blocking DA receptors decreases the reinforcing effects of many drugs (Wise and Bozarth, 1987; Johnson and North, 1992; Nutt et al., 2015; Volkow et al., 2017; Nestler and Lüscher, 2019; Solinas et al., 2019; Wise and Robble, 2020). The connection between DA and drug use is further supported by *in vivo* measurements of drug-evoked DA release in human and non-human animal studies (Hernandez et al., 1987; Chiara and Imperato, 1988; Robinson et al., 1988; Pontieri et al., 1995; Ito et al., 2002; Porrino et al., 2004; Volkow et al., 2006; Belin and Everitt, 2008; Willuhn et al., 2012, 2014). Humans with a history of drug use, including those meeting DSM criteria for SUDs, have altered DA system transmission and function (Stewart, 2008; Volkow et al., 2009; Diana, 2011; Leyton and Vezina, 2014; Ikemoto et al., 2015; Leyton, 2017). As such, popular theories of addiction and compulsive behavior are built on the notion of altered activity in the DA system (Robinson and Berridge, 1993; Everitt and Robbins, 2005; Wise, 2009; Keiflin and Janak, 2015; Nestler and Lüscher, 2019). Advances in neuroscience research technology and theory surrounding addiction-like behaviors in animal models of reward seeking have afforded the opportunity to characterize the role of precisely defined brain circuits and regions in behavior. In this review, we will discuss current evidence for regional and circuit-specific functions within the DA system in different aspects of addiction-like behavior, in the context of animal studies derived from DSM criteria for the behavioral features of SUDs.

## Dopamine Circuits

Most of the brain’s neurons are in two midbrain regions (Figure 1): the ventral tegmental area (VTA) and substantia nigra pars compacta (SNc). DA neurons in the VTA largely project to the ventral striatum, in particular, the nucleus accumbens (NAc) core and shell, comprising the mesostriatal pathway, and to other frontal targets in the pallidum, amygdala, and prefrontal cortex (Swanson, 1982; Ikemoto, 2007). Intermingled with DA neurons in the VTA are a substantial fraction of GABAergic and glutamatergic neurons (Olson and Nestler, 2007; Nair-Roberts et al., 2008; Bouarab et al., 2019). The SNc, in contrast, contains DA neurons that project almost exclusively to the dorsomedial (DMS) and dorsolateral (DLS) striatum, comprising the nigrostriatal pathway (Beckstead et al., 1979; Swanson, 1982; Fields et al., 2007; Ikemoto, 2007; Britt et al., 2012). At their targets in the striatum, DA neurons primarily contact GABAergic medium spiny neurons (MSNs) that contain excitatory type 1 (D1-MSNs), or inhibitory type 2 (D2-MSNs) DA receptors (Gerfen, 1984; Kupchik et al., 2015). Dopamine’s modulatory influence on striatal activity *via* these outputs is a predominant mechanism of behavioral control in reward learning and motivation. Notably, many drugs act in the striatum to increase DA release locally, *via* regionally specific terminal mechanisms (Collins and Saunders, 2020), which plays a key role in heterogeneous mechanisms of drug use, craving, and relapse behaviors that underlie features of SUDs (Koob and Bloom, 1988; Ahmed and Koob, 1998; Lobo et al., 2010; Thompson et al., 2010; Oliver et al., 2019).

Dopamine neurons across VTA and SNc circuits exhibit considerable heterogeneity with respect to behavioral function (Figure 1; Björklund and Dunnett, 2007; Lammel et al., 2014; Morales and Margolis, 2017; Cox and Witten, 2019; Collins and Saunders, 2020). In the classic framework, mesostriatal DA neurons contribute to learning and execution of goal-directed behaviors, while nigrostriatal DA, especially in the DLS, is involved in movement control and the execution of rigid, habitual actions (Haber et al., 2000; Hassani et al., 2001; Everitt, 2014; Burton et al., 2015; Saunders et al., 2018; Cox and Witten, 2019). From an extensive literature, deficits in VTA and SNc DA signaling typically impair learning and reward-directed behaviors, or movement planning, execution and vigor, respectively. Exaggerated VTA and SNc DA signaling, conversely, underlies compulsive motivation and behavioral inflexibility (Robinson and Berridge, 1993; Cardinal et al., 2002; Everitt and Robbins, 2005; Wise, 2005). In the context of Pavlovian learning, sensory cues associated with increased VTA DA neuron activity evoke approach behavior and acquire value that supports second-order conditioning of instrumental actions, which is critical for persistent and adaptive reward pursuit (Berridge, 2007; Flagel et al., 2011; Saunders and Robinson, 2012; Saunders et al., 2018). Nigrostriatal DA neurons, especially those projecting to the DMS, are important for linking instrumental actions with outcomes they produce (Yin et al., 2005). Further, activation of SNc DA neurons evokes movement, and their activity encodes movement initiation (Dodson et al., 2016; Coddington and Dudman, 2018; da Silva et al., 2018), suggesting they contribute more generally to movement invigoration. VTA DA neuron activity and release in the NAc is in contrast engaged when animals emit cue- or goal-directed movements (Carelli, 2004; Burton et al., 2015; Howe and Dombeck, 2016; Mohebi et al., 2019). As such, mesostriatal DA can be conceptualized as generating a motivational “pull” to cues and the rewards they predict, while nigrostriatal DA provides a “push” that underlies general behavioral invigoration or arousal (Bolles, 1967; Ostlund, 2019). Thus, while dissociable, normal activity in these parallel circuits is necessary for successful reward seeking and reward-based decision making (Arias-Carrión and Pǒppel, 2007; Aarts et al., 2011; Hsu et al., 2018; Le Heron et al., 2018, 2020; Cox and Witten, 2019; Collins and Saunders, 2020).

*Via* its roles in signaling expectation, value, and action invigoration, DA has strong influence on both goal-directed and habitual actions that result from reward learning. These fundamental behavioral classifications are each maladapted in addiction (Tiffany, 1990; Singer et al., 2018; Ostlund, 2019; Hogarth, 2020; Vandaele and Ahmed, 2021). Given that drugs impinge heavily on DA circuitry, functional, circuit-level differences within the DA system have important implications for the understanding of SUDs. In the following, we will review some of the ways in which mesostriatal and nigrostriatal DA pathways regulate behaviors associated with major SUD criteria. Notably, emerging work highlights that striatal DA is essential not only for features of SUDs characterized by exaggerated behavior, such as drug use and craving, but also for behavioral deficits, including impairments in decision making that underlie compulsive behavior, reduced sociality, and risk taking.

## Category I – Impaired Control of Substance Use

A hallmark of SUDs is a progression to impaired control over drug use, associated with increased drug intake, craving, and relapse vulnerability. As such, a major DSM criterion includes behavioral features such as “taking the substance in larger amounts or more often,” “spending a lot of time getting, using, or recovering from use of the substance,” and “cravings and urges to use the substance.” Animal studies of these SUD features are among the most common, leveraging the power of drug self administration models. The self administration models that align best with Category I SUD criteria are shown in Figure 1. Rodents, like humans, will readily self administer most commonly used drugs, such as; opioids, alcohol, cocaine, amphetamine, nicotine, and cannabinoids. In widely used paradigms, rats and mice are trained to engage in behaviors (typically, lever presses, or nose port responses) to receive drug doses delivered intravenously, orally, or *via* inhalation. Drug-associated cues and contexts, as well as small “priming” drug doses, stress and pain play a central role in promoting and maintaining drug use and relapse (Stewart, 1984; Balster and Lukas, 1985; Goeders and Guerin, 1994; Shaham et al., 2003; Chaudhri et al., 2008; Spanagel, 2017). In relapse models, the resumption of drug seeking following abstinence can be used as a behavioral index of drug “craving”. Notably, drug craving assessed in animal models undergoes “incubation” in the weeks to months following abstinence from many drug types. That is, the longer it has been since the last drug exposure, the greater the probability and intensity of relapse (Grimm et al., 2001; Venniro et al., 2016). This sensitization of the relapse-inducing power of drug cues in particular results in a persistent threat of a return to drug use, a feature of SUDs that is especially difficult to treat.

A major development in rodent addiction models came when it was discovered that giving rats extended access to drugs promotes an escalation of intake, where more drug is taken in a shorter time period, mimicking a central tenet of human SUDs. This has been observed with many drugs, including cocaine, heroin, methamphetamine, alcohol, and nicotine (Ahmed and Koob, 1998; Ahmed et al., 2000; Roberts et al., 2000; Kitamura et al., 2006; O’Dell et al., 2007). Escalation of drug intake following extended access is associated with other addiction-like features, including increased motivation for drug, drug seeking in the face of high effort cost, and seeking despite negative consequences. This approach has since informed a large portion of preclinical addiction research, including attempts to create a DSM-inspired composite addiction phenotype that can be applied to rodents (Deroche-Gamonet et al., 2004; Robinson, 2004; Vanderschuren and Everitt, 2004; O’Neal et al., 2020). More recently, intermittent access drug self administration models have gained attention (Zimmer et al., 2012; Kawa et al., 2019a). In these paradigms, brief periods of drug availability are interspersed with longer drug unavailability periods. This intermittency promotes rapid, binge-like drug intake that may better approximate some human drug use patterns. Intermittent self administration of cocaine, alcohol, and opioids, despite resulting in much less total drug intake compared to extended access models, promotes escalation of intake and elevated drug craving (Simms et al., 2008; Zimmer et al., 2012; Calipari et al., 2013; Kawa et al., 2016; O’Neal et al., 2020; Fragale et al., 2021; Samaha et al., 2021). Binge-like self administration can also develop in rats given extended, continuous access to cocaine, and individual differences in binge patterns early in self administration training predict the intensity of future use (Tornatzky and Miczek, 2000; Belin et al., 2009). Finally, some rodent models have also incorporated a behavioral economics framework to quantify drugs as commodities, to examine choice elasticity and demand (Oleson and Roberts, 2009; Bentzley et al., 2013; Mohammadkhani et al., 2019). This approach is useful for standardization of core behavioral indices related to decision making and motivation, which could facilitate quantitative comparisons across different tasks and reward modalities.

### Mesostriatal

Drugs act on DA circuits to promote synaptic plasticity that amplifies VTA activity and DA signaling in the NAc, even following a single exposure (Ungless et al., 2001; Mameli et al., 2009; Pascoli et al., 2012; Ungless and Grace, 2012; Ji et al., 2017; Morel et al., 2019; Thompson et al., 2021). Within the mesostriatal pathway, DA release evoked by drugs occurs *via* multiple mechanisms that impinge on VTA cells and their axon terminals (Ritz et al., 1987; Thomas et al., 2008; Volkow et al., 2009; Mark et al., 2011; Ford, 2014; Lammel et al., 2014). Given the central role that mesostriatal DA plays in reward learning and behavioral reinforcement (Fouriezos and Wise, 1976; Corbett and Wise, 1980; Witten et al., 2011; Steinberg et al., 2014), this system is key in the control of drug seeking, drug cue-evoked motivation, and craving. Manipulation of VTA DA neuron activity can regulate drug self administration in animal models. One way this has been studied is through manipulation of DA D2 autoreceptors. Activation of these receptors decreases activity of DA neurons and phasic DA release through a negative feedback loop (Schmitz et al., 2003), leading to changes in drug-taking behavior. Dopamine binding on VTA D2 receptors is negatively correlated with cocaine and amphetamine seeking and consumption (Buckholtz et al., 2010; Bello et al., 2011). Further, elevating VTA DA neuron activity *via* D2 knockdown increases cocaine self administration (Chen et al., 2018) and blocking the negative feedback activity of these receptors increases cocaine self administration (McCall et al., 2017). Additionally, rats with knockdown D2Rs will also work harder for sucrose and cocaine (de Jong et al., 2015). In line with this, reducing DA signaling in VTA neurons blunts cocaine self administration motivation, as measured by impaired behavior on a progressive ratio task (Ranaldi and Wise, 2001). These studies illustrate some ways that alterations in normal signaling with the mesostriatal pathway can alter motivation for drugs. Elevating VTA DA neuron activity can also promote impulsive choice behavior, for example, where rats prefer small, immediate rewards over larger rewards that require a longer waiting period (Bernosky-Smith et al., 2018), another component of impaired control over reward seeking behavior that is common in SUDs (de Wit, 2009; Dalley and Ersche, 2019).

Mesostriatal DA signaling is important for the escalation of drug intake. Repeated drug exposure, *via* passive administration or self administration, generally increases DA signaling in the NAc and produces exaggerated drug seeking motivation (Robinson and Berridge, 2001). Recent work, however, highlights how the pattern of drug intake can produce starkly different effects on mesostriatal DA circuits (reviewed in Samaha et al., 2021). Extended or long access to drug self administration, which produces escalation of drug intake, craving, and other addiction-like behaviors across a variety of drug types (Ahmed and Koob, 1998; Vanderschuren and Ahmed, 2013; Ahmed, 2018), is also associated with blunted drug-evoked NAc DA signaling, especially in cocaine use models (Mateo et al., 2005; Calipari et al., 2014; Willuhn et al., 2014; Siciliano et al., 2015). Intermittent, binge-like cocaine use, in contrast, sensitizes mesostriatal DA signals, relative to animals with a history of extended or continuous access, despite also producing strong escalation of intake and craving after much less total drug exposure (Kawa et al., 2019a). Intermittent drug exposure also selectively potentiates cocaine’s actions to inhibit DA transporter function, to facilitate elevated NAc DA signaling, relative to continuous or extended access (Calipari et al., 2013).

The distinction between the impact of extended versus intermittent access self administration on NAc DA signaling illustrates how DA circuits are sensitive to a number of features of drug experience, and careful consideration of the details of animal behavior models are critical for interpreting reported brain mechanism outcomes. For example, extended drug access may produce blunted NAc DA responses during well predicted and well learned drug-taking actions, an acute “hypodopaminergic” state that could promote greater drug taking to make up the reward deficit (Blum et al., 2000, 2015; Leyton and Vezina, 2014). Simultaneously, DA responses to drug-paired cues and unpredicted drug exposure can become sensitized, which underlies exaggerated cue-evoked drug seeking motivation, especially after a period of abstinence (Robinson and Berridge, 2001; Bradberry, 2007; Kawa et al., 2019b). Thus, it is possible for DA to be both down and upregulated in the context of SUD models, depending on specific DA circuits, task features, and when the signals are measured. Notably, humans use drugs in a variety of patterns, depending on the reason for use, the drug’s pharmacology and route of administration, and various other social and cultural factors (Gardner, 2011; Allain et al., 2015), so animal models featuring extended, continuous, and intermittent exposure are all likely important for capturing different features of addiction that reflect complex adaptations in the DA system.

Drug seeking responses maintained by the conditioned reinforcing value of cocaine-paired cues rely on VTA activity (McFarland and Kalivas, 2001; Shaham et al., 2003; Ciano and Everitt, 2004; Yun et al., 2004; Kufahl et al., 2009; Lüscher and Malenka, 2011; Mahler and Aston-Jones, 2012). This feature of mesostriatal control of addiction-like behavior is clear in models of relapse, which is often precipitated by exposure to a cue or location that was previously paired with drug delivery. VTA DA neurons mediate drug-cue induced relapse behaviors, and drug cues elicit VTA DA activity and DA release in the NAc with cocaine, alcohol, and other drugs (Ito et al., 2000; Phillips et al., 2003; Aragona et al., 2009; Ostlund et al., 2014; Wolf, 2016; Liu et al., 2020). Under periods of abstinence, VTA activity and NAC DA release facilitates relapse to cocaine, heroin, and alcohol in the presence of these cues (Shaham et al., 2003; Saunders et al., 2013; Corre et al., 2018; Mahler et al., 2019). Conversely, drug seeking is reduced by inactivation of the mesolimbic pathway (McFarland and Kalivas, 2001; Chaudhri et al., 2009; Saunders et al., 2013; Corre et al., 2018; Mahler et al., 2019; Valyear et al., 2020). These data fit within the framework of mesostriatal DA primarily controlling cue-guided or goal-directed drug seeking motivation.

### Nigrostriatal

A crucial part of SUDs is that drug taking is no longer recreational, but can become habitual, characterized by inflexible drug-taking actions that are insensitive to feedback and changing contingencies. A central feature of the organization of dopamine-striatum circuitry is the transition from ventromedial signaling early in reward learning, when behaviors are primarily goal directed, to later signaling in dorsolateral striatum that accompanies the development of habit-like behaviors (Haber et al., 2000; Joel et al., 2002; Ikemoto, 2007; Burton et al., 2015). This transition is readily demonstrated across drug classes in animal models (Zapata et al., 2010; Clemens et al., 2014; Hodebourg et al., 2019; Zhou et al., 2019), where habit-like behaviors are associated with nigrostriatal activity. Notably, the nigrostriatal DA pathway is less directly activated by acute drug exposure in animals with limited drug use history, compared to the mesostriatal pathway (Mereu et al., 1987; Ito et al., 2002; Keath et al., 2007; Belin and Everitt, 2008; Murray et al., 2012; Willuhn et al., 2012, 2014). Drug use is thought to accelerate the transition to addiction-like behaviors *via* progressive engagement of the dorsolateral striatum. Evidence from rodent self-administration models supports this notion. Dopamine signaling in response to cocaine and cocaine-associated cues is initially strongest in the NAc as rats learn to self administer the drug. Over time, the DA response to cocaine delivery in the NAc weakens, and DLS DA signaling emerges (Ito et al., 2002; Willuhn et al., 2012, 2014). Further, the emergence of robust DLS DA signaling predicts the degree of escalation of drug use, and DA signaling and activity in the DLS is necessary for robust cocaine and alcohol self administration only after extended drug use (Belin and Everitt, 2008; Corbit et al., 2012; Murray et al., 2012; Willuhn et al., 2012, 2014). In line with this, DLS D1-MSN activity is associated with escalated methamphetamine self-administration (Oliver et al., 2019). Extended nicotine self administration is associated with exaggerated neural activity in the SNc and DLS (Clemens et al., 2014). DLS DA signaling is necessary for cocaine self administration maintained on second-order reinforcement schedules (Vanderschuren et al., 2005), which is thought to reflect the development of stimulus-response associations that are resistant to extinction. Further, within the DLS, well learned alcohol seeking actions are preferentially encoded over drug receipt (Fanelli et al., 2013), which is consistent with the notion of this system in mediating habitual or ritualistic features of drug taking (Everitt and Robbins, 2005). Together these results suggest that the nigrostriatal DA pathway is recruited to promote the escalation of drug use and rigid drug-intake patterns, which underlies the development of addiction-like states in SUDs.

An inability to change behavior in response to changing outcome value is proposed to be a key reason behind drug craving and the draw of drug-associated cues. Interestingly, SNc DA neurons have been shown to encode reward values over the long term, even when these rewards are no longer expected (Kim et al., 2015). This could suggest that even if tolerance to some of the pharmacological features of a drug is developed, SNc DA retains the drug taking “habit” *via* an inflexible memory of the reward when first experienced. Disordered memory could also impact relapse susceptibility. For example, inflating rewarding memories of drug use, or decreasing memories of negative experience, could make one more likely to use a drug even after a period of abstinence. Supporting this general notion, in one study where rat SNc DA neurons were chemically lesioned, lesioned rats performed worse on a task that delivered negative feedback for poor performance (Da Cunha et al., 2001). Exaggerated or otherwise altered nigrostriatal activity that accompanies drug exposure may produce a state of feedback insensitivity that promotes exaggerated behaviors in SUDs.

## Category II – Impaired Social Behavior

The social and lifestyle consequences of SUDs are perhaps the most difficult to study in non-human animals, as this category includes behavioral features such as “continuing to use, even when it causes problems in relationships” and “giving up important social, occupational, or recreational activities because of substance use”. Common models of social behavior that align with SUD diagnostic criteria are shown in Figure 1. Recent modeling efforts have focused on elements of sociality that are readily measured in species like rodents, including social interaction and affiliative and rearing behaviors, and the effects of social experience on decision making. Importantly, the interaction between social experience and drug-related behaviors is bidirectional. Conspecific-based stressors, including disrupted parental care, social isolation, and social defeat or subordinate status, generally increase future self administration of amphetamine, cocaine, alcohol, and heroin (Schenk et al., 1987; Bardo et al., 2013). In contrast, positively valenced or rewarding social interactions can be protective against cocaine and heroin self administration, craving, and other addiction-like behaviors, even in rats with extensive drug-taking experience (Banks and Negus, 2017; Venniro et al., 2018, 2019). Social play is highly rewarding in most mammals and relies on normal function in striatal DA systems (Vanderschuren et al., 1997; Manduca et al., 2016). As such, social behavior can be disrupted with prenatal or adolescent drug exposure (Trezza et al., 2014; Achterberg et al., 2019). Further, isolation from social play, particularly during adolescence, can promote future drug use and decision-making deficits associated with addiction (McCutcheon and Marinelli, 2009; Lesscher et al., 2015). Like social exposure, other forms of environmental enrichment and access to other non-food rewards, such as an exercise wheel, can have protective effects against escalation of cocaine self administration (Zlebnik and Carroll, 2015). While much remains to identify neural mechanisms of these effects, this work potentially underscores the importance of prosocial, lifestyle, and community-based SUD treatments for humans (Higgins et al., 2003; Meyers et al., 2011; Stitzer et al., 2011).

### Mesostriatal

Given its core role in reward processes, the mesostriatal pathway has a central role in social behavior. Social interaction increases mesostriatal DA signaling and NAc neurons are active during approach to both novel conspecifics and pair-bonded partners (Robinson et al., 2002; Gunaydin et al., 2014; Scribner et al., 2020). Mesostriatal DA neurons projecting to the NAc are necessary for normal social interaction behavior (Gunaydin et al., 2014). Inhibition of mesostriatal DA neurons can disrupt exploration of novel conspecifics (Bariselli et al., 2018) and stimulating these neurons can enhance social preference (Bariselli et al., 2016). Drug-evoked changes in VTA DA signaling and physiology can impact these social behaviors. For example, neonatal exposure to amphetamine increases VTA DA activity and decreases social behavior in adulthood (Fukushiro et al., 2015). This may be due to a D1-specific mechanism in the NAc, as blocking D1-like DA receptors in this region rescues impaired social bonding behavior in amphetamine-treated male prairie voles (Liu et al., 2010). Further, following repeated exposure to amphetamine, female prairie voles show decreased social bonding behavior, accompanied by decreased DA D2 receptor immunoreactivity and increased DA levels in the NAc. Notably, administering oxytocin can restore social bonding and NAc DA levels, suggesting an interaction between oxytocin and DA systems in social behavior and drug use (Young et al., 2014).

In addition to drugs affecting mesostriatal DA signaling and social behavior, social interactions can in turn alter drug taking and signaling as well. Social defeat stress has been shown to enhance the long-term potentiation of glutamatergic signaling in the VTA as well as potentiate cocaine conditioned place preference (Stelly et al., 2016). Further, increased cocaine use following social defeat stress can be mimicked by directly infusing corticotropin releasing factor into the VTA, which modulates DA neurons (Leonard et al., 2017). Maternal separation can also disrupt reward seeking and DA signaling in the mesostriatal pathway. For example, female, but not male mice subjected to maternal separation and social isolation show a decreased conditioned place preference for a palatable reward and a decreased level of D1 receptor mRNA in the NAc (Sasagawa et al., 2017). Social isolation can also reduce the total dendritic length of MSNs in the NAc (Wang et al., 2012), while increasing DA signaling (Hall et al., 1999; Yorgason et al., 2016). The ability to produce aberrant or exaggerated mesostriatal DA signaling is thus one mechanism by which social stressors can produce addiction vulnerability.

### Nigrostriatal

Given its role in movement control, the function of nigrostriatal DA in the context of Parkinson’s disease (PD) models has driven most research investigations. Notably, while severe PD is primarily characterized by motor impairments, patients also experience cognitive and emotional deficits that affect social behavior. As such, some (Tadaiesky et al., 2008; Matheus et al., 2016) suggest this pathway could be critical for social impairments seen in people with SUD’s. Supporting this, in rats, nigrostriatal damage can increase depression-like symptoms and cognitive impairments in a social recognition test, as well as promote social withdrawal (Tadaiesky et al., 2008; Matheus et al., 2016). Interestingly, these effects were observed after an initial anhedonic response which mapped onto changes in dorsal striatal D1 and D2 receptor activity. Specifically, DA lesions increased the density of D1 and D2 receptors in the DLS after 7 days, which returned to control levels at 21 days when the anhedonic-like effects were no longer present and social withdrawal emerged. Cholinergic interneurons (ChIs) in the dorsal striatum, which can regulate DA release locally *via* terminal mechanisms (Collins and Saunders, 2020), may also play a role in regulating social behaviors in mild nigrostriatal lesioned mice. For example, inhibiting striatal ChIs reverses social memory impairments caused by DA depletion (Ztaou et al., 2018). While the connection between nigrostriatal DA and socially-based behavioral changes in the context of SUDs has not been characterized in animal models, this system is engaged by social experiences (Robinson et al., 2002). Lesions of the substantia nigra in general seem to reduce some social behaviors, including mate investigation and social grooming (Eison et al., 1977). Other studies have shown that rats with SNc lesions exhibit no difference on a social interaction test (Loiodice et al., 2019), however, so the connection between nigrostriatal DA signaling and normal social behaviors, independent of non-specific motor effects, requires more consideration.

Rearing conditions also affect the nigrostriatal dopaminergic pathway. Social enrichment can reverse the behavioral effects of nigrostriatal lesions in mice. Specifically, it slows the progressive nature of lesioning damage as well as reverses motor impairments (Goldberg et al., 2012). Social isolation increases DA release and uptake in the dorsal striatum in rats, *via* alterations in DA transporter function, which results in greater psychostimulant potency (Yorgason et al., 2016). Together these studies show that disruptions to the nigrostriatal dopaminergic pathway produce social and cognitive deficits and different social conditions can affect this pathway and in turn, drug reactivity.

## Category III – Risky Substance Use

The DSM criteria for risky use of substances includes “using substances again and again, even when it puts you in danger” and “continuing to use, even when you know you have a physical or psychological problem that could have been caused or made worse by the substance”. Common decision-making tasks thought to capture these SUD criteria are shown in Figure 1. Recently, animal models of behaviors related to this SUD criterion have become more common, including “risky” choice assessment, conflict procedures, and punishment-resistant intake models (Venniro et al., 2020).

Risky substance use is associated with compulsivity, which in animal models is typically operationalized as a continuation of behavior despite negative consequences. This is measured in a few ways. For example, rodent tasks that approximate human gambling conditions can assess cost-benefit decision making, sensitivity to loss or punishment, and performance under conditions of uncertainty as metrics of risk taking (Orsini et al., 2015; Winstanley and Clark, 2016; Lüscher et al., 2020). Approach-avoidance paradigms impose a situation of motivational conflict on the research subject, between the urge to seek out a reward and avoid an aversive or costly stimulus (Oleson and Cheer, 2013). In related punishment-based models, reward seeking actions also result in the delivery of noxious or otherwise aversive stimulus, such as footshock, or a bitter taste. A history of escalated use of several drugs, including cocaine, alcohol, and opiates promotes punishment resistance (Vanderschuren and Everitt, 2004; Pelloux et al., 2007; Marchant et al., 2013; Hopf and Lesscher, 2014; Blackwood et al., 2020; Monroe and Radke, 2021; Domi et al., 2021). Notably, these tasks are particularly useful for assessing individual differences in addiction-like behavior, as only a subset of animal subjects will persist in drug seeking in the face of high cost (Shaham et al., 2003; Everitt and Robbins, 2005; Cooper et al., 2007; Vanderschuren et al., 2017; Giuliano et al., 2018).

### Mesostriatal

Ventral tegmental area DA neuron stimulation, in the absence of other reward-related stimuli, can lead to compulsive-like behavior. When given the option to self stimulate VTA DA neurons in the face of a punishing footshock, a subset of mice will perseverate, enduring high shock levels (Pascoli et al., 2015). Mesostriatal DA signaling is also important for conflict-based behaviors. In rats, phasic DA signaling in the NAc encodes motivational conflict: cues signaling threats evoke greater DA release compared to neutral cues, and this signal correlates with successful behavioral avoidance (Oleson et al., 2012; Gentry et al., 2016). Further, blocking NAc DA abolishes, and increasing NAc DA potentiates, drug-seeking behavior in a task where rats were required to cross an electrified barrier to receive infusions of cocaine (Saunders et al., 2013). Notably, this effect was strongest in the subset of rats who were willing to experience the highest shock levels, suggesting a link between mesostriatal DA and individual differences in motivation in the face of adverse consequences.

Disruptions to the mesostriatal pathway modulate so-called risk-based decisions that incorporate cost-benefit probabilities (Orsini et al., 2015). For example, DA signaling within the NAc encodes risky decisions in gambling-inspired tasks (Sugam et al., 2012). Notably, subsets of VTA DA neurons have different roles in risky decision making (Verharen et al., 2018). Activation of the mesostriatal pathway reduces sensitivity to loss and punishment, while activating the VTA-PFC pathway promotes risky decisions when there is no loss present. Further, stimulating the VTA after a non-rewarded risky choice, which overrides the phasic dip in DA release that would normally occur, biases rats to choose a risky reward in the future and reduces sensitivity to reward omissions (Stopper et al., 2014).

Hyperdopaminergic states, such as those evoked by drugs, can lead to disordered decision making that favors risk taking, feedback insensitivity, and behavioral inflexibility (Stalnaker et al., 2009; Izquierdo et al., 2010; Groman et al., 2018). Drug exposure can affect future risk-based decisions, *via* alterations in mesostriatal DA. For example, adolescent alcohol exposure in rats reduces overall mesostriatal DA tone, but potentiates phasic DA release, an effect that positively correlates with risk preference, and is reversed when the DA signal is normalized (Schindler et al., 2016). Adolescent drug exposure selectively disrupts NAc DA encoding of costs (Nasrallah et al., 2011), suggesting that feedback insensitivity associated with addiction-like behavior relies on specific drug-induced mesostriatal adaptations. Interestingly, the role of mesostriatal DA in risky decision making may be somewhat sex dependent, as males exhibit decreased cue-induced risky choice behavior following VTA inhibition while females exhibit increased risky choice (Hynes et al., 2021). These results suggest that altering VTA DA activity leads to an impairment of decision making that is facilitated by, and could contribute to, risky drug use.

Outside of the striatum, VTA projections to the prefrontal cortex also play a major role in reward seeking in risky situations. During reward-seeking actions, risk of punishment diminishes synchrony between the VTA and PFC (Park and Moghaddam, 2017). Further, during learning, phasic activity in the PFC of rats encodes risky seeking actions but not safe taking actions or reward delivery, suggesting that the PFC is preferentially involved in the learning of punishment probability. This effect was also sex specific, with females exhibiting greater sensitivity to probabilistic punishment than males (Jacobs and Moghaddam, 2020). However, this effect may not be mediated by VTA-PFC DA projections *per se*, given some studies showing that these neurons do not exhibit differential activity under threat of punishment (Verharen et al., 2020).

### Nigrostriatal

The role of nigrostriatal DA in habit-like actions underlies its connection to compulsive behaviors that contribute to risky drug use (Everitt and Robbins, 2005). One characteristic of habit-like behavior is an encoding of a stable reward value despite changes to the reward itself. Notably, a subset of DA neurons in the lateral SNC demonstrate a “sustain-type” firing pattern that is insensitive to changes in expected reward after extended learning (Kim et al., 2015). Further, DLS DA axon terminals don’t exhibit a clear decrease in activity when the actual reward is smaller than predicted, unlike terminals in dorsomedial and ventral striatum (Tsutsui-Kimura et al., 2020). This lack of feedback within the nigrostriatal pathway in learning could result in drug-taking behavior despite a negative consequence or in risky situations.

In an approach-avoidance decision making task, dopamine’s actions within the DMS have opposing effects on behavior, with D1-MSN activation facilitating approach and D2-MSN receptors suppress approach. In contrast, DLS DA manipulations don’t as clearly affect approach-avoidance behavior (Nguyen et al., 2019). However, after extended access to cocaine self administration, DLS inactivation selectively reduces self administration in the face of punishment, compared to unpunished use (Jonkman et al., 2012). Further, individual differences in the extent to which alcohol seeking engages activity in the DLS predicts susceptibility to punishment resistance (Giuliano et al., 2019), suggesting a specific role in compulsivity and threat-based feedback. DMS inactivation increases risky choice on a probabilistic discounting task in rats, suggesting that it in contrast can facilitate flexibility in reward prediction (Schumacher et al., 2021). Taken together, these results are consistent with the notion that SNc-DLS DA projections contribute to inflexible behavior, the SNc-DMS projections promote flexibility in goal-directed behavior, both of which are engaged during risk-reward decisions (Lerner et al., 2015; Vandaele and Ahmed, 2021). Recent evidence suggests that unlike other DA neurons, projections to the caudal “tail” portion of the dorsal striatum preferentially encode threatening stimuli and threat avoidance, relative to positively valenced stimuli (Menegas et al., 2018). This suggests they could have a critical role in risk-based decisions and compulsivity. Notably, the effect of a history of drug exposure on nigrostriatal and striatal tail function in conflict or avoidance tasks remains relatively unexplored. Nevertheless, the above data suggest that in SUD patients, dysregulation or imbalance of DA signaling across SNC output targets could promote risk insensitivity to underlie dangerous substance use.

Further insight into the connection between altered nigrostriatal DA signaling and compulsive behavior comes from PD patients receiving DA replacement therapy, which can result in impulse control disorders that lead to risky decision making. Levodopa treatment, for example, can be effective at restoring motor function associated with nigrostriatal DA degeneration in Parkinson’s, but a subset of patients experience an increase in addiction-like behaviors, including compulsive use of levodopa itself (Lawrence et al., 2003; Evans et al., 2006). These effects are partially mediated by the emergence of D1-receptor supersensitivity that results from nigrostriatal DA neuron degeneration (Gerfen, 2003). Notably, and consistent with the human PD phenomenon, a subset of parkinsonian rats display high sensitivity to DA replacement drugs, after a history of drug self administration (Engeln et al., 2013). More work on the link between Parkinson’s states and the behavioral effects of DA replacement in animal models will be useful in understanding nigrostriatal DA’s role in risky and disordered drug use (Cenci et al., 2015; Napier et al., 2020).

## Other Considerations for Animal Models of Substance Use Disorder

### Drug Type and Route of Administration

Given historical patterns in addiction research, much of the current conceptualization of SUD is based on behavioral modeling in a relatively narrow range of compounds, compared to the broad scope of drug types and routes of administration used by humans. Much of the work discussed here, for example, made use of stimulant drugs (primarily cocaine), although application of SUD animal models to non-stimulant drugs is becoming more common. This is an important consideration for future research, given that the majority of drug use in contemporary humans is of non-stimulant drugs, including opioids, cannabis, and alcohol (Substance Abuse and Mental Health Services Administration, 2019). More SUD-model research on a broader set of drugs is critical because not all drugs engage the same learning mechanisms to produce patterns of addiction-like behaviors. Nicotine, for example, is relatively weak as a primary reinforcer of drug self administration, compared to other drugs (Pontieri et al., 1996). Instead, nicotine may augment reward seeking to promote addiction-like behavior by potentiating the motivational value of other stimuli and actions *via* non-associative mechanisms (Donny et al., 2003; Chaudhri et al., 2006). This heterogeneity is underscored by the fact that while most self administered drugs increase DA release and act on striatal circuits, they do so to different degrees, and through different mechanisms that may produce unique signaling patterns with specific behavioral relevance (Wise and Bozarth, 1987; Johnson and North, 1992; Nutt et al., 2015; Volkow et al., 2017; Nestler and Lüscher, 2019; Solinas et al., 2019; Wise and Robble, 2020). Further complications come from the fact that some drugs used by humans, such as some hallucinogens, do not as reliably increase DA signaling, and are not readily self administered by rodents, making the application of SUD behavioral models described here difficult (Griffiths et al., 1979; Fantegrossi et al., 2008; Serra et al., 2015).

Drugs are taken by humans through different routes of administration, including most commonly *via* oral consumption, intravenous or subcutaneous injection, or inhalation. The route of delivery affects the pharmacological impact that drugs have on the brain and peripheral physiology, producing unique neurobiological changes and vulnerabilities to addiction-like behaviors (Jones, 1990; Gossop et al., 1992; Cone, 1998; Allain et al., 2015). Intravenous injection and smoking produce the fastest rise and highest drug concentration in the blood, which is associated with greater DA signaling and neural activity in reward circuits (Samaha et al., 2021). Notably, the large majority of drug self administration animal models have relied on intravenous or oral consumption drug delivery, which may obscure unique neurobiological and behavioral adaptations produced by other delivery routes. Smoked cannabis and nicotine are among the most consumed drugs by humans, for example, underscoring the need for SUD models that are amenable to inhalation exposure. Recent work has progressed on this front, with technology for vapor-based delivery of cannabis, cannabinoids, nicotine, and other drugs in rodents (Nguyen et al., 2016; Marusich et al., 2019; Freels et al., 2020).

### Co-substance/Poly-Drug Use Models

Simultaneous or serial use of multiple drug types is a common feature of human behavior and is reflected in many SUD patients. Alcohol, nicotine, and cannabis are commonly used alongside drugs like cocaine and heroin, for example, and poly-use SUD patients experience worse treatment outcomes, compared to patients who primarily use a single drug (Leri et al., 2003; Mccabe et al., 2006; Substance Abuse and Mental Health Services Administration, 2019; Crummy et al., 2020; Compton et al., 2021). Despite this, animal models of SUDs have nearly exclusively made use of single-drug procedures, studying drug effects in isolation. Given the unique adaptations in the DA system produced by different drug types, this single-drug focus likely prevents understanding of unique brain changes associated with poly-drug use. From a treatment perspective, a given individual’s specific drug combination history could produce individualized SUD vulnerabilities that are not captured in classic models. Recently, more emphasis has gone to modeling poly-drug use in rodents (reviewed in Crummy et al., 2020). For example, rats will readily self administer some drug cocktails, including cocaine and heroin (Crombag and Shaham, 2002). This produces DA responses in the NAc that are greater than those evoked by either drug alone (Hemby et al., 1999). In line with this, exposure to both methamphetamine and morphine results in greater locomotor activity than either drug in isolation (Trujillo et al., 2011), and sequential self administration of alcohol and cocaine produces unique neuroadaptations in the NAc compared to cocaine alone (Stennett et al., 2020). Thus, some single-drug studies may actually produce below threshold neurobiological changes, resulting in failure to detect SUD-like features that are more common in poly-use humans. Other work has been done on drug combinations that are popular among humans, such as alcohol and nicotine. Access to both of these can have synergistic effects on reward-related behaviors in rodents, although individual preferences for one drug or the other may drive co-self administration competition (DeBaker et al., 2020; Angelyn et al., 2021). Poly-drug studies can also offer insight into unique, drug-specific pathways to addiction-like behavior and treatment. For example, social defeat stress more reliably produces escalation of speedball self administration, compared to heroin self administration (Cruz et al., 2011). Further, methadone treatment, commonly used in human opioid use patients, is effective at reducing both cocaine and heroin relapse in rat models (Leri et al., 2003). Notably, little is known about unique adaptations in nigrostriatal DA circuits associated with poly-drug use. If some drug combinations evoke greater DA release in the NAc compared to others, they may also produce exaggerated SNc DA activity, facilitating a more rapid transition to habit-like drug related behaviors.

### Individual Differences in Substance Use Disorder Vulnerability

Variability in drug use profiles is highlighted by the fact that among all recreational drug users, only ∼20% progress to meet some DSM diagnostic criteria of SUD (Substance Abuse and Mental Health Services Administration, 2019). Furthermore, in the current framework, a SUD diagnosis requires the presence of only two or more criteria (Figure 1). While more severe cases of SUD typically involve several common criteria (American Psychiatric Association, 2013), mild to moderate cases can present with relatively divergent behavioral features. This creates challenges for treatment, as there is no singular “addiction phenotype”: the SUD of one person can look quite different from another person. Animal studies have underscored this by demonstrating that only a small fraction of rodents, when given access to drugs, will progress to develop multiple addiction-like behavioral criteria (Deroche-Gamonet et al., 2004; Robinson, 2004; Vanderschuren and Everitt, 2004; O’Neal et al., 2020). Further, and perhaps more striking, when given the choice between a drug reward and a non-drug reward, such as food or a mate, only a small fraction of rats choose the drug option (Lenoir et al., 2007; Cantin et al., 2010). This non-drug preference persists even in rats with a history of extended drug self administration (Caprioli et al., 2015), but can be overcome with high drug doses and in tasks that equate the rate of reinforcement for drug and non-drug rewards, at least for cocaine (Thomsen et al., 2013; Beckmann et al., 2019). This suggests that individual differences in drug metabolism may intersect with and impact decision making in the context of drug use, contributing to individual vulnerabilities to SUD. This represents a challenge for animal models, where the comparatively limited genetic diversity of research subjects may elide some variability factors. Recent work has made use of “heterogeneous stock” rodents – the product of multiple strain crossings - to increase genetic diversity and investigate individual differences in addiction-like behavior and reward learning in the context of drug use (Hughson et al., 2019; de Guglielmo et al., 2021; King et al., 2021; Sedighim et al., 2021). One such study identified a relationship between gut bacterial content and behavioral features of impulsivity and attention (Peterson et al., 2020). Thus, individualized peripheral systems such as the microbiome can impinge on central brain systems, including DA circuits, in ways that could produce unique SUD vulnerabilities (Lucerne et al., 2021).

Humans exhibit considerable individual differences in SUD characteristics as a function of sex, gender, age, and social and environmental demographics (Brady and Randall, 1999; Degenhardt et al., 2017; Substance Abuse and Mental Health Services Administration, 2019). The factors that promote exaggerated drug use in only a fraction of people are likely myriad, but individual differences in DA system activity and function play a key role (Piray et al., 2010; Saunders et al., 2013). For example, humans with genetic polymorphisms that result in elevated DA system activity show greater reward cue-evoked striatal activity and craving that could denote a predisposition toward exaggerated drug use (Wittmann et al., 2013; Ray et al., 2014). Consistent with this, in animal models, variability in DA signaling and expression of striatal DA receptors is associated with higher drug cue responsivity and relapse (Flagel et al., 2007; Verheij and Cools, 2008; Piray et al., 2010; Saunders et al., 2013; Klanker et al., 2015; Ferguson et al., 2020). Furthermore, at certain stages of the estrus cycle, female rodents have larger NAc DA signals in response to cocaine and cocaine-associated cues, which is thought to underlie their generally higher propensity for addiction-like behaviors (Becker and Cha, 1989; Becker, 1999; Calipari et al., 2017; Johnson et al., 2019).

Given the circuit-specific DA functions in reward learning and addiction-like behavior, some of which we have outlined here, a detailed appreciation of the anatomical locus of variability within DA systems in humans will be essential to forming a link to unique SUD-related vulnerabilities. This will require a better understanding of the DA system across multiple levels of analysis, from genetic and developmental trajectories to *in vivo* circuit connectivity and activity patterns. Critically, much work remains to better understand how the larger mesocorticostriatal network changes over time as both an antecedent and consequence of drug taking. A functional circuit diagram, coupled with computational approaches for modeling preclinical individual and sex-based differences in decision making strategies (Groman et al., 2019; Chen et al., 2021), will be important for determining the neurobehavioral mechanisms underlying unique vulnerabilities for different types of SUD.

## Conclusion

Here we have reviewed evidence for overlapping, but distinct mesostriatal and nigrostriatal DA circuit functions in behavioral outcomes that are relevant to addictions and SUDs (summarized in Figure 1). Dopamine innervation to the striatum contributes to multiple, parallel functions in the context of addiction-like behavior, with the mesostriatal pathway providing a “pull” toward drug seeking by signaling drug and drug-associated stimulus value, especially early in the use cycle. The nigrostriatal pathway, and particularly DLS projecting DA neurons, in contrast are more important for generating the “push” toward exaggerated drug use by controlling rigid, feedback insensitive drug-seeking actions. Notably, as highlighted above, striatal DA is important not only for these positive symptom features of SUDs, including exaggerated seeking and craving, but also for impairments in decision making that underlie compulsive behavior, reduced sociality, and risk taking. Through the use of animal models, greater understanding of the functional heterogeneity of the DA system and related networks can offer insight into this complex symptomatology and may lead to more targeted treatments.

## Author Contributions

CP and LE conceptualized and wrote the original manuscript draft, in consultation with BS. CP and BS generated Figure 1. All authors contributed to writing and editing the final manuscript and approved its submission.

## Conflict of Interest

The authors declare that the research was conducted in the absence of any commercial or financial relationships that could be construed as a potential conflict of interest.

## Publisher’s Note

All claims expressed in this article are solely those of the authors and do not necessarily represent those of their affiliated organizations, or those of the publisher, the editors and the reviewers. Any product that may be evaluated in this article, or claim that may be made by its manufacturer, is not guaranteed or endorsed by the publisher.
